# Routine immunization experience and practices during the COVID-19 pandemic of caregivers attending a tertiary hospital in Cape Town

**DOI:** 10.3389/frhs.2023.1242796

**Published:** 2023-11-01

**Authors:** A. Manan, T. Wessels, H. Finlayson

**Affiliations:** Department of Paediatrics and Child Health, Stellenbosch University, Cape Town, South Africa

**Keywords:** childhood immunizations, immunization practices, caregiver experiences, COVID-19, pandemic

## Abstract

**Introduction:**

Immunizations are successful, cost-effective interventions for the control of infectious diseases and preventing mortality. Lockdown restrictions during the COVID-19 pandemic had adverse effects on child-health including access to immunizations. Our study aimed to document immunization status, describe caregiver experiences around accessing immunizations during the COVID-19 pandemic and identify any significant factors associated with immunization status.

**Methods:**

Caregivers, with children between the ages of 10 to 33 months, attending Tygerberg Hospital Paediatric Department were invited to complete an anonymous survey from 15th September–15th December 2022. Data was captured using a REDCap questionnaire and analysed using Stata Version 17.

**Results:**

171 caregivers completed the survey. Immunizations were up to date in 81%. Most (155, 88%) agreed it was important to immunize their child. A third of caregivers (55) felt it was unsafe to attend the clinic and 37% (62) agreed it was difficult to attend. Caregivers receiving a social grant (*p* = 0.023) or who felt safe attending clinic (*p* = 0.053) were more likely to be up to date with immunizations. Three-quarters (128, 78%) were aware of recommendations to continue immunization. These caregivers were more likely to think it was important to immunize on time (*p* = 0.003) and to receive family encouragement (*p* = 0.001). Caregivers were more likely to attend clinic if they felt it was important to vaccinate on time (*p* < 0.001) or felt safe attending clinic (*p* = 0.036).

**Conclusion:**

Immunization rates were higher than expected but below global targets. Although caregivers feel immunizations are important, unknowns still instilled fear of attending clinics. Social factors such as family support and social grants improve vaccine seeking behaviour.

## 1. Introduction

Immunizations are recognized globally as one of the most successful and cost-effective interventions for the control of infectious diseases. Annually immunizations prevent 2–3 million deaths in children under five years of age (1, 2). They improve health equity across low-, middle- and high-income countries by reducing disability and mortality (3). Immunization programs have been implemented worldwide to ensure immunizations at every contact with the healthcare system (4, 5).

Fifty-six percent of global deaths in children under five years of age occur in Africa (1, 3), many of which could be prevented by immunizations. While overall global immunization rates reach 86%, Africa lags behind with the lowest rate of 76% (1, 3). In South Africa, the 2016 Demographic and Health Survey (DHS) revealed that up to 40% of the country's children are not fully immunized for their age (5).

The COVID-19 pandemic increased the burden on healthcare systems and had severe consequences related to rigid lockdown restrictions, social distancing, and prolonged school closures resulting in adverse effects on the pediatric population (6, 7). One of the consequences of lockdown restrictions was a decrease in childhood immunization rates. A recent systematic review showed a relative median decline of 11%, affecting upper and lower -middle income countries (decline of 14% and 18% respectively) more than low-income countries (decline of 3%) (8). It is estimated that during 2020, 30 million children missed their third diphtheria-tetanus-pertussis vaccine, and 27 million children missed their measles vaccines (9). The United States and Singapore documented a drop in measles vaccination, whilst 40 million children in Pakistan missed their polio immunization due to the cessation of vaccination campaigns in April 2020 (10–12).

In South Africa various levels of public restrictions were implemented from March 2020. These levels varied according to the prevalence of COVID-19 infections and included evening curfews, restrictions of public events and gatherings as well as alcohol and tobacco sales. Mask wearing was compulsory, and citizens were requested to limit movement and only leave their house for emergencies. Government recommendations were that childhood vaccination should continue (13). Despite this South Africa also reported a decrease in immunization rates. The National Department of Health reported a decrease in national immunization coverage from 82% in April 2019 to 61% in April 2020 during high levels of restriction. Most concerning was the sharp decrease in second dose measles vaccine coverage rates from 77% in April 2019 to 55% in April 2020 with the Western Cape Province dropping to a low of 48% during that period (14).

Reasons for low immunization rates prior to the COVID-19 pandemic have been well documented and are largely related to sociodemographic factors, including extremes of maternal age, limited education, single parents, and low family income (1). Limited parental knowledge about immunization benefits is the most frequently reported factor that influenced childhood immunization uptake (1, 3, 5). In South Africa lack of parental awareness of immunization schedules, inadequate training of healthcare workers and the high workload of women have been identified as negative influencers of immunization coverage (15, 16).

Fear of acquiring COVID-19 infections has been reported as a significant factor behind falling immunization rates during the pandemic (17–20). Whilst parental perspectives on the importance and effectiveness of childhood immunizations remained unchanged, they experienced many barriers during lockdown periods that influenced their motivation and the opportunity to vaccinate their children (18, 19, 21). These barriers included uncertainties about operational hours of clinics as well as uncertainties around booking vaccination appointments (18, 19).

Whilst there has been data published on parental experiences and perceptions around accessing immunizations during the COVID-19 pandemic in other countries, none have been published in the South African setting. This study aimed to document immunization rates within our setting and describe caregiver experiences around accessing immunizations during the COVID-19 pandemic. In addition, it aimed to identify any risk factors pertaining to a lack of knowledge of government recommendations as well as immunizations not being up to date.

## 2. Materials and methods

This was an explorative descriptive study undertaken at Tygerberg Hospital, a tertiary referral hospital in the Cape Town metropole. The paediatric department provides varying levels of care to half of the Western Cape Province's paediatric population <14 years, estimated at 787 000 in 2016 (22). Approximately 8 500 admissions and 1 400 ambulatory patients were seen in the wards surveyed during 2022 (23).

We surveyed caregivers with children between the ages of 10 and 33 months attending the emergency, ambulatory services and paediatric wards over a 3-month period starting on 15 September to 15 December 2022. Age was calculated to include children requiring immunization during the COVID-19 pandemic starting from March 2020 to December 2021, when lockdown restrictions were eased. Caregivers of children admitted to high care and intensive care areas were excluded due to severity of disease and parental concern.

Data was collected by trained medical students or the principal investigator using an anonymous structured questionnaire administered electronically in the language of their choice to caregivers accompanying their children to the wards. A REDCap survey link provided access to the questionnaire and data was saved automatically.

### 2.1. Data collection tool

The anonymous survey consisted of four sections: compulsory informed consent; a research questionnaire consisting of demographic details such as age, sex, education, employment status and family size; questions centred around immunization experiences and perceptions which were structured in a 5-point Likert question format (strongly agree to strongly disagree) and finally information around immunization status obtained from the road to health booklet (RTHB). The RTHB is a handheld booklet given at birth to all caregivers in South Africa to record their child's growth parameters, receipt of immunizations and other healthcare interventions.

The questionnaire was adapted for our South African setting from a previously validated questionnaire which assessed the impact of COVID-19 lockdown on immunization behaviour in caregivers living in London (Supplementary Data Sheet S1). Consent for use was obtained from the author (19).

### 2.2. Sample size calculation and data analysis

Sample size was calculated based on the reported data suggesting a drop in the second-dose measles coverage in the Western Cape to 48% (14). A sample size of 160 participants was required to achieve a desired precision of ±8% for a 95% confidence interval. Sample size estimation was done using WINPEPI (www.brixtonhealth.com/pepi4windows.html).

Data was extracted from REDCap and analysed using Stata 17 (College Station, Texas 77845 USA). For associations the Likert score was condensed for ease of analysis into agree (strongly agree and agree) and did not agree (neutral, disagree, strongly disagree) for ease of analysis.

Continuous variables were summarised using mean (standard deviation) and compared using a t-test. Categorical variables were summarised using count (percent) and compared using chi-squared test or Fisher's exact test. We report immunization rate with the corresponding 95% confidence interval. Immunizations up to date were defined as children who had received all their vaccinations on time during the pandemic as well as those who had delayed immunizations but had now caught up the required schedule for their age. A *p* value <0.05 was considered statistically significant.

We tested the association between binary outcome variables and exposure of interest using univariate and multivariate binomial regression and reported relative risks as measures of association.

Approval from the Human Research Ethics Committee (HREC) at Stellenbosch University was obtained. HREC S22/06/013_COVID-19.

## 3. Results

171 caregivers completed the survey over the three-month period. There were 10 incomplete responses but entered data was included in the analysis. Demographic details of caregivers are shown in Table 1. The majority 159 (93%) of caregivers were female and had a mean age of 30.4 (±8.3) years. One hundred and forty-five (88%) caregivers had their RTHB present, and 132, 81% (95% CI 74%–86%) children's immunizations were up to date at the time of the study (Table 1). Three quarters (128, 78%) of caregivers were aware of the government recommendation to continue routine immunizations during the COVID-19 pandemic.

**Table 1:** Demographic details of caregivers and immunization status of children.

		*n* (%)
Sex (*n* = 171)	Female	159 (93.02%)
	Male	12 (6.98%)
Residence (*n* = 171)	Within Cape Metro	144 (84.21%)
	Outside Cape Metro	27 (15.79%)
Level of Education (*n* = 170)	Junior (Grade 1–7)	9 (5.29%)
	High School (Grade 8–11)	76 (44.71%)
	Grade 12	64 (37.65%)
	Tertiary diploma or degree	21 (12.35%)
Relationship Status (*n* = 168)	With a partner	100 (59.52%)
	Single parent	46 (27.38%)
	Raising my grandchild	14 (8.33%)
	Raising a family member	3 (1.79%)
	Foster parent	2 (1.19%)
	Other	3 (1.79%)
Employment (*n* = 168)	Fulltime	48 (28.57%)
	Part-time	29 (17.26%)
	Unemployed with partner support	43 (25.60%)
	Unemployed with family support	23 (13.69%)
	Unemployed with no support	11 (6.55%)
	Other	14 (8.33%)
Government Grant (*n* = 168)	Yes	99 (58.93%)
	No	69 (41.07%)
Number of Children (*n* = 168)	One	50 (29.76%)
	Two	61 (36.32%)
	Three	37 (22.02%)
	Four or more	20 (11.90%)
RTHB present (*n* = 164)	Yes	145 (88.4%)
	No	145 (88.4%)
Immunisations status (*n* = 162)	Always up to date	129 (79.63%)
	Delayed but catch up complete	3 (1.85%)
	Delayed catch up in progress	10 (6.17%)
	Not up to date	17 (10.49%)
	Unable to recall	3 (1.85%)

RTHB, road to health booklet.

### 3.1. Caregiver experiences

**Figure 1:**
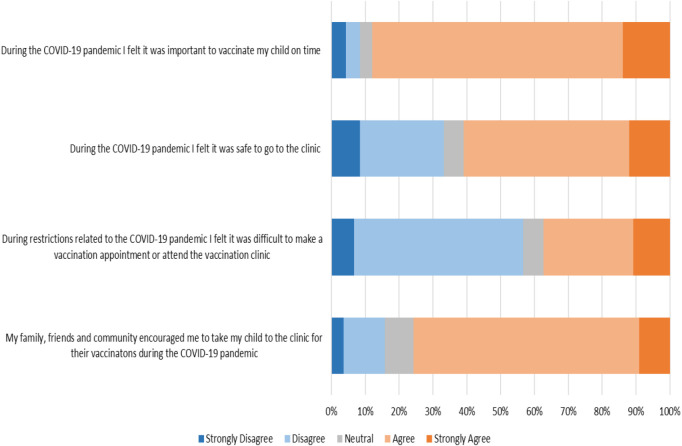
Likert responses of caregivers’ experiences during the COVID-19 pandemic.

Caregiver experiences during the COVID-19 pandemic and periods of restriction are shown in Figure 1. Caregiver experiences were mostly positive with 153 (88%) of caregivers who either agreed or strongly agreed that it was important to take their child to the clinic for immunizations. Caregivers felt that family members encouraged them to take their child to the clinic, 125 (75%) agreed or strongly agreed with the statement. A third of patients (55, 33%) did not feel it was safe to attend the clinic and 62 (37%) either agreed or strongly agreed that it was difficult to make an appointment or attend the vaccination clinic over the lockdown period.

### 3.2. Risk factors for immunization

When trying to identify risk factors for immunizations not being up to date, we looked at demographic factors, caregiver experiences, knowledge of governmental recommendations and whether the RTHB was present (Table 2). Receiving a government support grant was the only significant factor, caregivers receiving a grant were more likely to be up to date with immunizations than those who did not (79, 88% vs. 50, 72%; *p* = 0.023). Other factors associated with immunizations being up to date was caregivers who felt safe to attend the clinic (*p* = 0.053), caregivers with lower education level (*p* = 0.055) and caregivers with less than four children (*p* = 0.052), these however did not reach significance.

**Table 2:** Risk factors for not having immunizations up to date.

		Immunisations up to date		
		No *n* (%)	Yes *n* (%)	*p*-Value
Age (years)	Mean (std.deviation)	29.9 (±5.4)	30 (±8.2)	0.860
Sex	Male	1 (8.33%)	11 (1.670%)	0.466
	Female	29 (19.72%)	118 (80.20%)	
No. of children	One	5 (10.20%)	44 (89.80%)	0.052
	Two	13 (22.41%)	45 (77.59%)	
	Three	3 (8.82%)	31 (91.18%)	
	Four/more	6 (33.33%)	12 (66.67%)	
Education	School	20 (14.49%)	118 (85.51%)	0.055
	Diploma/Postgraduate	7 (33.30%)	14 (66.67%)	
Relationship status	Raising child with partner	15 (15.79%)	80 (84.21%)	0.416
	Single parent	10 (22.73%)	34 (77,27%)	
	Raising someone else's child (grandchild, foster etc.)	2 (10.00%)	18 (90.00%)	
Employment	Employed	14 (18.92%)	60 (81.08%)	0.544
	Unemployed	13 (15.29%)	72 (84.71%)	
Government Grant	Yes	11 (12.22&)	79 (87.78%)	**0** **.** **023**
	No	19 (27.54%)	50 (72.46%)	
Area of residence	Inside Metro	22 (16.54%)	111 (83.46%)	0.463
	Outside Metro	5 (19.23%)	21 (80.77%)	
Aware of Recommendation	Aware	21 (17.07%)	102 (82.93%)	1
	Not Aware	6 (16.67%)	30 (83.33%)	
Felt safe to go to the clinic	Disagree	15 (24.19%)	47 (75.81%)	0.053
	Agree	12 (12.37%)	85 (87.63%)	
Important to Immunise on time	Disagree	3 (15.00%)	17 (85.00%)	1
	Agree	24 (17.27%)	115 (82.73%)	
Difficult to make an appointment	Disagree	14 (14.00%)	86 (86.00%)	0.192
	Agree	13 (22.03%)	46 (77.97%)	
Encouraged by Family	Disagree	7 (18.92%)	30 (81.08%)	0.803
	Agree	20 (16.39%)	102 (83.61%)	
Has RTHB with them	Yes	24 (16.90%)	118 (83.10%)	1
	No	3 (17.65%)	14 (82.35%)	

RTHB, road to health booklet.

*P*-values in bold indicate reached significance.

Receiving a social support grant reach significance on both univariate, relative risk 1.2 (95% CI 1.02–1.42) and multivariate analysis, after adjusting for age, gender, and employment status, RR 1.22 (95% CI 1.05 to 1.42), *p* = 0.001.

### 3.3. Influence of caregiver experiences on taking children to the clinic

Caregivers who agreed that it was important to immunize on time were more likely take their children to the clinic (139, 97% vs. 12, 60% *p* < 0.000). Those caregivers who felt safe were also more likely to take their child to the clinic (95, 96% vs. 56, 86% *p* = 0.036). Other caregiver experiences did not influence whether they took their child to the clinic (Table 3).

**Table 3:** Experiences, perceptions and if they took the child to the clinic.

	Took child to the clinic			
		Yes	No	*p*-value
Parents felt safe	Disagree	56 (86.15%)	9 (13.85%)	**0** **.** **036**
	Agree	95 (95.96%)	4 (4.00%)	
Important to vaccinate on time	Disagree	12 (60%)	8 (40.00%)	**0** **.** **000**
	Agree	139 (96.53%)	5 (3.47%)	
Encouraged by family	Disagree	33 (84.62%)	6 (15.38%)	0.082
	Agree	151 (92.07)	13 (7.93%)	
Difficult to make an appointment	Disagree	97 (94.17%)	6 (5.83%)	0.236
	Agree	54 (88.52%)	7 (11.48%)	
Aware of Government Recommendation	Yes	120 (93.75%	8 (6.25%)	0.162
	No	31 (86.11%)	5 (13.89%)	

*P*-values in bold indicate reached significance.

### 3.4. Influence of knowledge of government recommendations

Caregivers who felt it was important to immunize their children on time and who received family encouragement were more likely to know the government recommendations to continue vaccination (*p* = 0.003 and *p* = 0.001). Other factors did not reach significance (Supplementary Table S1).

Only 13 (7%) of caregivers did not attempt to go to the clinic during the COVID-19 pandemic. Over half of these caregivers (10, 60%) reported feeling scared of contracting COVID-19. Half (7, 53%) of the caregivers who did not attend the clinic caught up their immunizations after restrictions were lifted. Seventy percent (5) of these caregivers agreed that it was easy to start the catch-up process at the clinic. Only 5 (50%) caregivers who did not catch up responded to the reasons they felt it was difficult, of these 3 caregivers (60%) felt the waiting queue was too long, the rest did not disclose reasons for difficulty catching up immunizations.

## 4. Discussion

To our knowledge, this is the first study that describes routine immunization experiences and practices of South African caregivers during the COVID-19 pandemic.

We found that 81% of caregiver's immunizations were up to date which was higher than the Western Cape provincial average of 48% for measles vaccinations, as reported in the press during the beginning of the pandemic (14). Our results are in keeping with other studies which found that disruptions to childhood immunizations were higher in the first months of the pandemic and normalized towards the end of 2020 (5, 8, 23). Data from the Western Cape Provincial Annual Health report documents immunization coverage under one year of age in 2019/2020 as 82.2% and 2020/2021 as 82.9% (24), showing that overall immunization rates were maintained in the province during the pandemic.

The service team responsible for the Expanded Programme on Immunizations (EPI) maintained immunization coverage even with the added COVID-19 challenges. This was after the Western Cape implemented strategies to improve the immunization services at the facilities and to enhance safety for caregivers by minimising the risk of acquiring infection. These measures included appointment systems to minimise waiting time and the creation of secondary sites where “healthy” clients could receive preventative services such as immunizations (Sonia Botha, Provincial EPI co-ordinator, 24/05/23) (25).

The Western Cape EPI task team undertook various campaigns to maintain immunization rates and services within the province. These included regular printed media as well as social media and radio adverts highlighting the importance of attendance and immunizations. The public-private partnerships were enhanced, and child health services were prioritized and protected (25).

Other strategies included recalling caregivers and outreach for catch up-immunizations with assistance of community-based services (Sonia Botha, Provincial EPI co-ordinator, 24/05/23) (25). These strategies are likely to have contributed to the high rate of awareness of the government recommendations to continue vaccination during the lockdown period. More than three-quarters of caregivers were aware of the recommendations, which is in keeping with data from the United Kingdom where 74.4% of survey respondents were aware of their national recommendations to continue routine vaccination practices (19).

Despite our higher-than-expected immunization rates, it is important to note that these figures still fail to meet the global EPI targets of 90% nor the Western Cape EPI targets of 86% (24). The recent measles outbreak in South Africa which started in October 2022 suggests that immunization rates remain suboptimal (26). Strengthening of EPI services is needed via improved healthcare strategies to raise awareness and promote access to vaccinations.

### 4.1. Caregiver experiences

#### 4.1.1. Safety

A third of caregivers in our study did not feel it was safe to attend the clinic. A systematic review examining reasons for reduced uptake of routine immunizations in low-middle income countries, reported fear of contracting the COVID-19, was the primary reason affecting health seeking behavior (27). Eighty percent of caregivers in India and 61% of Saudi Arabian caregivers reported fear of contracting COVID-19 during the pandemic (27, 28). High income countries including the UK and the USA also reported that parental hesitancy to visit immunization facilities was due to perceived fear and risk of acquiring COVID-19 infection (17–20).

As expected, there was a trend that caregivers who felt safe to attend the clinic were more likely to have their children's immunizations up to date although this did not reach statistical significance. Mishra et al. found that 83% of caregivers in Eastern India felt that safety was more important than vaccination (27). Although 72% of survey respondents in England felt it was safe to attend the clinic, these caregivers reported to have delayed immunizations initially but once attending the facility reported positive experiences (19). In these specific studies safety measures were in place, such as screens between patients, social distancing, donning of protective gear by staff and the availability of hand sanitisers.

Prior to the pandemic and currently, there are various safety concerns of caregivers and their children attending primary care facilities in South Africa. These range from exposure to other infectious agents such as Tuberculosis, the lack of child friendly spaces as well as exposure to violence in the community (29). Thus, it is imperative to prioritise the safety of caregivers and children, in order to improve access to immunizations and other primary health care services.

#### 4.1.2. Importance

Over 80% caregivers agreed that it was important to take their child to the clinic for immunizations on time, 85% of parents in the UK felt similarly (19). Literature suggests that parental perspectives on the importance of immunizations remained the same before and after the pandemic (18, 19, 21, 30). Caregivers deliberately delayed routine immunization out of fear of exposure to COVID-19 infection, rather than a change of attitude towards vaccination (17, 18, 20). Caregivers understood the importance of vaccinations in preventing disease, but it was weighed up against perceived risks of contracting the COVID-19 virus. While there was fear of acquiring COVID-19, caregivers felt that acquiring a vaccine preventable disease would be less likely during periods of restriction as children were isolated from others (19).

We did not look at specific barriers that influenced caregiver motivation to immunize their children however over a third strongly agreed that it was difficult to make an appointment or attend the immunization clinics over the lockdown period. These have previously been reported in the UK and Saudi Arabia as barriers to accessing immunizations (18, 19).

Family encouragement to take children to the clinic during the lockdown period was high. It is evident that family plays a role in decision making around seeking health services. A study in Indonesia showed that lack of support from parents, husbands, and friends led to caregivers not seeking to provide complete primary immunizations to their children (31). Family support and encouragement are critical factors for enabling completion of the immunization schedule. In South Africa, cultural norms are that the family participates in caring for and raising a child, thus having a great influence on decisions around immunizations (31). Forty one percent of households in South Africa are headed by women, with the lack of partner support particularly identified as a reason for children missing immunizations (5, 15, 32). Supportive partners can greatly enhance knowledge around immunizations as partners jointly improve health seeking behaviour for their offspring (1, 31).

### 4.2. Factors associated with immunization rates

Caregivers who received social support grants were more likely to be up to date with immunizations on multivariate analysis. South African children under the age of 18 qualify for a social support grant that is paid to the primary caregiver provided they pass the means test (33). The means test determines whether a person qualifies to receive a grant by evaluating income and assets (33). Social support grants have been shown to increase likelihood of clinic visits for monitoring of weight, nutrition, and health (34). They help alleviate poverty and improve nutritional and health outcomes as grants are spent on food, education, and basic services. This is especially impactful in female-headed households (35, 36). Caregivers responsible for children who qualify for a social support grant should be encouraged to apply to improve quality of life and healthcare outcomes.

Family size has previously been shown to influence immunization with support for both large and small families having better immunization rates (1, 37). Our study did not reach significance but there was a trend that families with four or more children were less likely to be immunized, supporting the notion that parents of larger families may have more daily tasks causing missed vaccinations (18, 28, 38). Our study showed that there was a trend that caregivers with post-secondary school education were less likely to have immunizations up to date. This is in contrast to other studies in Africa which showed that parents with at least a primary or secondary school education, were more likely to fully immunize their children compared to parents with no formal education (1, 3, 5). We did not look specifically at no education in our study and post-secondary education numbers were low. Caregivers with a diploma and postgraduate degrees may have better access to growing social media influence on vaccine hesitancy and therefore choose to not take children for routine immunizations. An online survey conducted in China showed that parents with higher education levels were more likely to hesitate to immunize their children against COVID-19 (39). In India and Chennai there was increased vaccine hesitancy among parents belonging to an educated population, social media and television was the source for vaccine-related misinformation (40).

There was no difference in immunization rates within or outside the metro despite previous studies suggesting that distance to clinic may influence immunization rates (6). The Western Cape Province has an efficient community-based service in remote areas which includes home visits and encouragement of good healthcare practices and routine immunizations in children (41). These services may help improve immunization rates in hard to reach areas. There was no difference in immunization status according to knowledge of government recommendations around immunizations, previous studies showed increased immunization rates in those that knew the recommendations (19, 23). Parents in the UK were more likely to be aware of the government recommendation after an announcement by the public health emergency unit later in 2020 (19). Research by the South African department of Planning, Monitoring and Evaluation in 2021 highlights deep levels of distrust by the public in Government and public services (42) Despite this, regular presidential press statements and social media campaigns raised awareness around the pandemic regulations (43). Caregiver recall may have been influenced by the timing of our study which took place towards the end of the pandemic.

Few caregivers answered questions around not catching up their child's immunizations and we were therefore unable to make informed inferences, however caregivers who attempted catch up after delayed immunization felt that long waiting queues made the process difficult. This was previously identified as a barrier to routine immunization uptake (44).

## 5. Limitations

Our study has a number of limitations. The sample size was calculated from data at the time of the pandemic which suggested that immunization rates had dropped significantly, considering the higher vaccination rates we found, a larger sample size may have provided more accurate results.

This study was undertaken two years after the start of the COVID-19 pandemic, which may have recall bias from caregivers who had forgotten true perceptions at the time of COVID-19 lockdown. Although the study was done at a hospital which provides all levels of care, these caregivers were already in a healthcare facility and may have better health seeking behaviours than caregivers in the community leading to an inflated immunization rate.

Despite these limitations our immunization rate was similar to the official rates reported by the provincial healthcare systems. Lastly, we used a quantitative survey to evaluate caregiver perceptions, the addition of a qualitative component may have given additional insights into parental perceptions and experiences.

## 6. Conclusion

We found an immunization rate of 81% which was higher than expected but below global targets. Although caregivers may feel that immunizations are important, unknown factors such as the COVID-19 pandemic may still instill fear of attending clinics, steps should be taken to mitigate perceived dangers at primary care facilities. Social factors such as family support and access to a social grant are likely to improve immunization seeking behaviour. There should be focussed efforts on improving social support of caregivers as well as providing clear information on clinic activities. Further studies examining caregivers' perceptions and practices when accessing routine immunization are needed to address lack of knowledge around immunization services and guide improved immunization targets.

## Data Availability

The raw data supporting the conclusions of this article will be made available by the authors, without undue reservation.
