# Supplementary material for: Routine immunization experience and practices during the COVID-19 pandemic of caregivers attending a tertiary hospital in Cape Town
Source: Front Health Serv. 2023 Nov 1;3:1242796. doi: 10.3389/frhs.2023.1242796 (PMC10646605; doi:10.3389/frhs.2023.1242796)
Supplement: Supplementary file 1 [file Table1.docx]

Supplementary Table 1: Factors influencing knowledge of Government recommendations

|  |  | Government Recommendation | | p-value |
| --- | --- | --- | --- | --- |
|  |  | yes | no |  |
| Education | School | 114(79.72%) | 29(20.28%) | 0.256 |
|  | Diploma/Postgraduate | 14(66.67%) | 7(33.3%) |  |
| Area Of residence | Within Metro | 108(78.26%) | 30(21.74%) | 1 |
|  | Outside Of the Metro | 20(76.92%) | 6(23.08%) |  |
| Sex | Male | 10(83.3%) | 2(16.67%) | 1 |
|  | Female | 118(77.63%) | 34(22.37%) |  |
| Circumstances | Raising a child with a partner | 80(81.63%) | 18(18.37%) | 0.369 |
|  | Single parent | 33(73.33%) | 12(26.67%) |  |
|  | Raising Someone Else’s child | 15(71.43%) | 6(28.57%) |  |
| **Experiences** |  |  |  |  |
| Felt it was safe to attend the clinic | Disagree | 46(70.77%) | 19(29.23%) | 0.083 |
|  | Agree | 82(82.83%) | 17(17.1%) |  |
| Important to immunise on Time | Disagree | 10(50.0%) | 10(50.0%) | **0.003** |
|  | Agree | 118(81.94%) | 26(18.06%) |  |
| Difficult to make an appointment | Disagree | 82(79.61%) | 21(20.39%) | 0.562 |
|  | Agree | 46(75.41%) | 15(24.59%) |  |
| Receive Family Encouragement | Disagree | 22(56.41%) | 17(43.59%) | **0.001** |
|  | Agree | 106(84.80%) | 19(15.20%) |  |
| Receiving Government Grant | Disagree | 71(74.74%) | 24(25.26%) | 0.256 |
|  | Agree | 57(82.61%) | 12(17.39%) |  |
