# Supplementary material for: Routine immunization experience and practices during the COVID-19 pandemic of caregivers attending a tertiary hospital in Cape Town
Source: Front Health Serv. 2023 Nov 1;3:1242796. doi: 10.3389/frhs.2023.1242796 (PMC10646605; doi:10.3389/frhs.2023.1242796)
Supplement: Supplementary file 2 [file Datasheet1.docx]

Supplementary Materials : Fig.2 Anonymous questionnaire used for survey

ROUTINE IMMUNISATION PRACTICES OF PARENTS/CAREGIVERS DURING THE COVID-19 PANDEMIC.

Please read the information below about the survey you are being asked to complete

ROUTINE IMMUNISATION PRACTICES OF PARENTS/CAREGIVERS DURING THE COVID-19 PANDEMIC.

By clicking I consent to take the survey you are confirming that you are:

- over 18 years old.
- have read and understood the above explanation about the study; and
- you agree to participate

You also understand that your participation in this study is strictly voluntary.
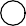
 I consent to taking the survey

Tick the Option you choose for anonymous data sharing with journals:

I agree to have my anonymous information shared with journals during publication of results of this study. I do not agree to have my anonymous data shared with journals during publication of results of this study.


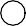

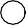


Sex assigned at birth male


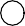

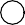


female

What is your age in years

I currently live within the Cape Town Metro

outside of the Cape Town Metro


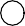

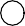


My highest level of education is Grade 1-7 Grade 8 -11

Grade 12


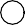

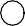

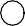

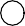


Post Grade 12 course or degree

Which of the following best describe your current I am raising a child with a partner circumstances I am a single parent

I am raising my grandchild


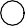

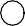

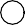

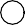

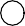

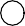


I am raising a family member

I am a foster parent (unrelated to the child) Other

Which of the following best describes your I am working full time

circumstances in the last year I am working part time


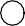

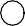

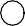

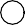

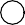

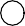


I am unemployed but supported by a spouse/partner I am unemployed but supported by my parent

I am unemployed with no support from family/partner Other


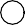

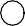


I get a child support grant (R480 SASSA grant) yes no

How many children are you the parent/guardian of 1

2


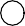

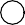

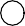

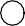


3

4 or more

The age of my first child in months is

the age of my second child in months is

The age of my third child in months is

Number of my fourth child in months is

During the coronavirus (COVID-19) pandemic I felt it was important to vaccinate my child/children on time for their routine vaccinations


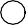
 Strongly disagree
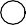
 Disagree
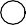
 Neither agree or disagree
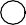
 Agree
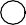
 Strongly Agree

During the COVID-19 pandemic I felt it was safe to go to the clinic to vaccinate my child/children on time for their routine vaccinations


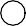
 Strongly disagree
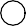
 Disagree
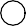
 Neither agree or disagree
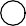
 Agree
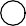
 Strongly Agree

During restrictions related to the COVID-19 pandemic I felt it was difficult to make a vaccination appointment or attend the vaccination clinic


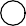
 Strongly disagree
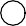
 Disagree
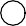
 Neither agree or disagree
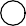
 Agree
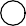
 Strongly Agree

My family, friends and community encouraged me to take my child/children to the local clinic for their routine vaccinatons during the COVID-19 pandemic


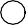
 Strongly disagree
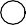
 Disagree
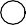
 Neither agree or disagree
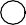
 Agree
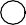
 Strongly Agree

The recommendation from the South African Government is that childhood immunisations should continue as normal during the COVID-19 pandemic.

Are you aware of this recommendation?
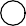
 yes
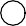
 no


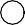

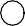


During the COVID-19 pandemic since the strict lockdown yes in March 2020 did you try take you child to the clinic no to get vaccinated

I did not take my child to the clinic for vaccinations I was scared I may get sick with coronavirus during the COVID-19 pandemic because I did not have enough money

I did not have any transport


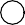

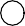

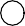

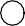

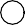

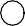


I though the clinic was closed I worked extra shifts

Other

Although I missed vaccinations during the COVID-19 yes

pandemic. I took my child to catch up his/her no vaccination when COVID-19 cases were low


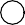

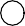


It was easy to get the catch up vaccination when I took my child to the clinic


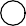
 Strongly disagree
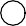
 Disagree
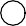
 Neither agree or disagree
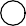
 Agree
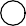
 Strongly Agree

Things that made taking my child for catch up vaccinations difficult included

I was turned away from the clinic many times There was a long waiting queue

The clinic was out of stock of the vaccination I needed

There was not enough staff at the clinic The clinic was closed

other

I have my road to health booklet with me today yes no


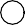

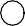


**You have now finished your part of the survey. Please hand the tablet to the research**

**facilitator (together with you road to health booklet) who will complete the last question.**

RTHB information filled by researcher Immunisations always up to date immunisations currently not up to date immunisations delayed and catch up complete immunisations and catch up in process


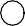

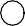

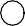

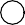

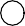


Unable to recall
